# Detecting a Canal of Nuck Hydrocoele in a Child with a Ventriculoperitoneal Shunt Using POCUS

**DOI:** 10.24908/pocusj.v10i02.19411

**Published:** 2025-11-17

**Authors:** David J. McCreary, Maria Munir, Milan Gopal

**Affiliations:** 1 Paediatric Emergency Department, Sunderland Royal Hospital, Sunderland and South Tyneside NHS Foundation Trust; 2 Department of Paediatric Surgery, Great North Children's Hospital, Newcastle upon Tyne

**Keywords:** POCUS, VP shunt, CSF pseudocyst, Canal of Nuck

## Abstract

In females, failure of the processus vaginalis to close properly can result in continued outpouching of the parietal peritoneum through the inguinal canal into the labia majora, forming a structure known as the Canal of Nuck. In rare cases, a Canal of Nuck hydrocoele can develop in association with the presence of a ventriculoperitoneal (VP) shunt, leading to symptoms of pain and discomfort in the inguinal region. We present the first reported case of a Canal of Nuck hydrocoele identified using POCUS in a child. This case highlights the role of POCUS as a safe, reliable, first-line imaging tool for identifying Canal of Nuck hydrocoeles in patients with VP shunts.

## Case history

**Figure 1.**
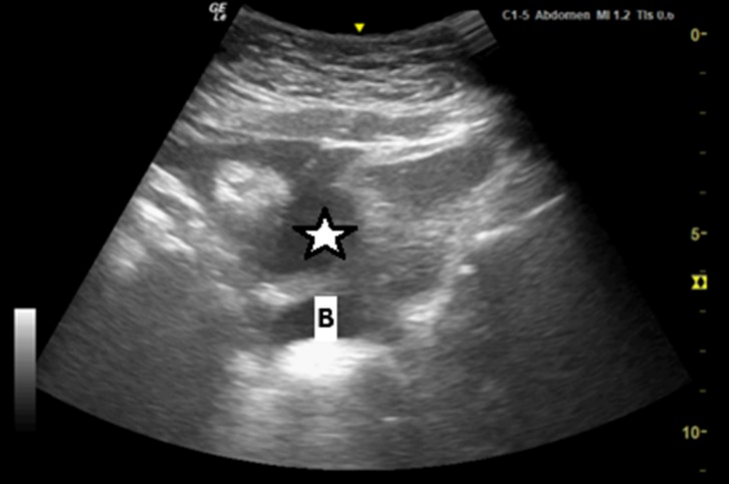
Point of care ultrasound (POCUS) image of pelvis in transverse plane using the curvilinear 5-12 MHz probe, demonstrating a moderate amount of free fluid (*) anterior to the bladder (B).

**Figure 2.**
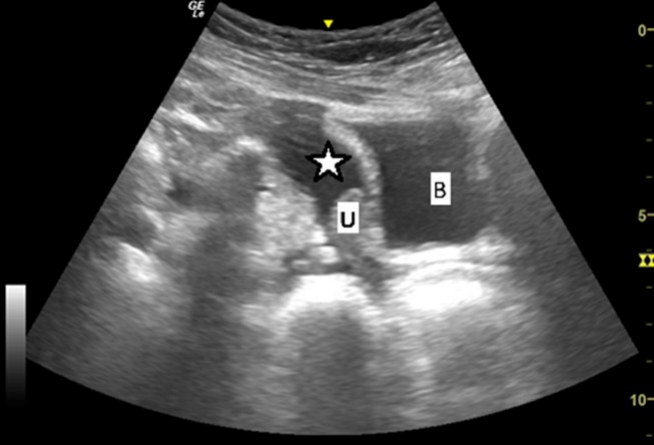
Point of care ultrasound (POCUS) image of pelvis in longitudinal plane using the curvilinear 5-12 MHz probe. This demonstrates a moderate amount of free fluid (*) in the pelvis. The bladder (B) is located inferior to this, and the uterus (U) is shown.

**Figure 3.**
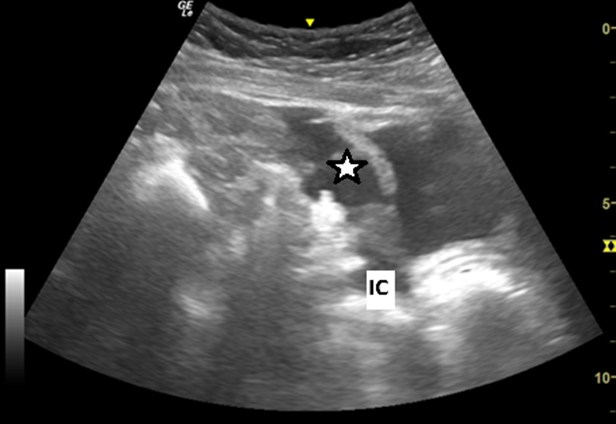
Point of care ultrasound (POCUS) image of the right pelvis and upper inguinal region in the longitudinal/oblique plane using the curvilinear 5-12 MHz probe. This demonstrates free fluid (*) in the pelvis communicating with the right inguinal canal (IC)

**Figure 4.**
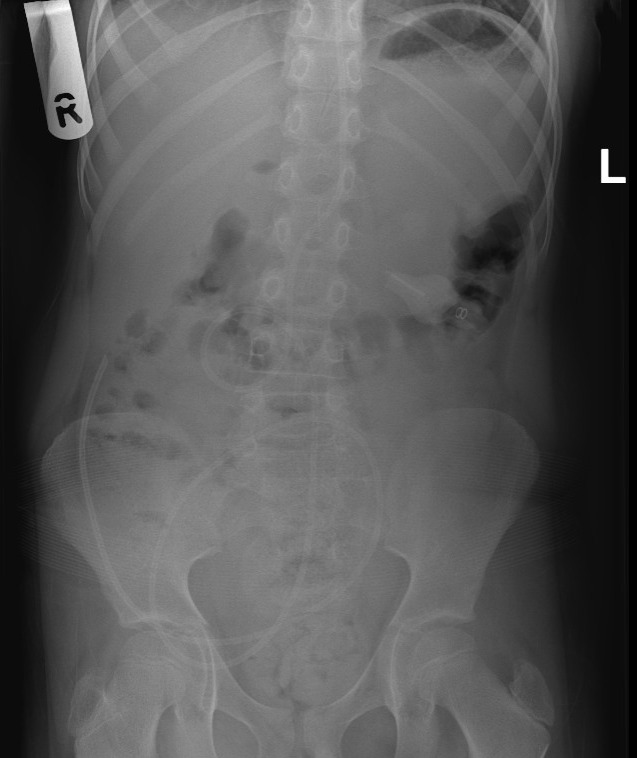
Abdominal X-ray demonstrating ventriculoperitoneal shunt and location of its tip.

**Figure 5.**
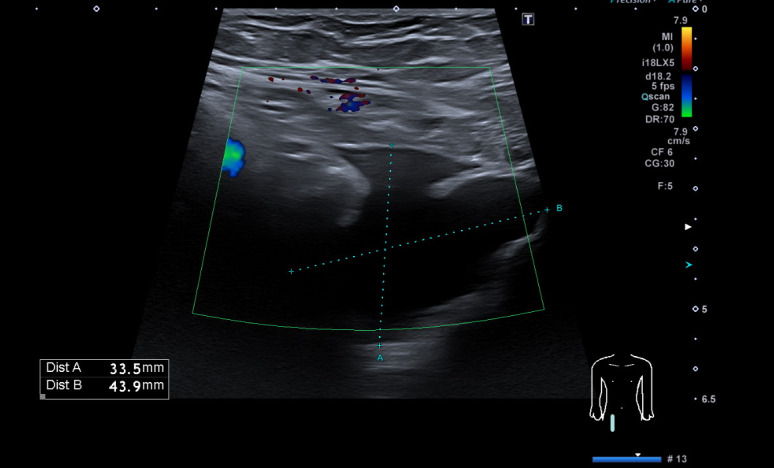
Radiology performed ultrasound of the pelvis demonstrating free fluid in the right inguinal region. No enhancing flow pattern is seen on colour Doppler.

**Figure 6.**
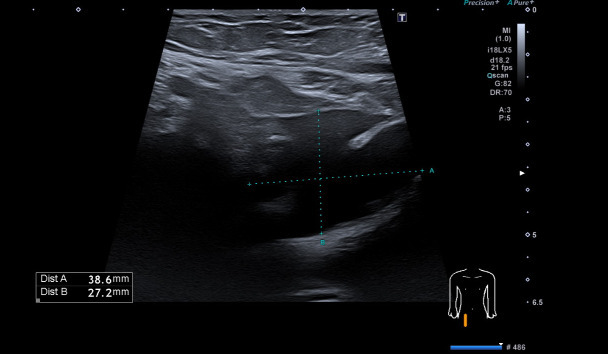
Radiology performed ultrasound of the pelvis demonstrating free fluid in the right inguinal region.

A 7-year-old girl presented to the paediatric emergency department with discomfort in her right groin. She was born prematurely at 27+6 weeks and developed communicating hydrocephalus for which she had a ventriculoperitoneal (VP) shunt inserted as an infant. She had no other systemic symptoms and appeared well. Examination revealed mild tenderness in her right iliac fossa but no obvious swelling. A POCUS examination was conducted, which revealed a significant amount of free fluid evident in the transverse and longitudinal planes throughout the pelvis. This suggested fluid migration more distally into the inguinal canal (See Figures 1-3). The appearances in the right iliac fossa did not reveal any signs consistent with appendicitis or any other surgical abdominal pathology that would account for the free fluid. Due to concern about the possibility of migration of the distal end of her VP shunt, an abdominal X-ray was taken, as shown in Figure 4. This confirmed that the shunt tip was in the expected location. A radiology departmental ultrasound was requested, which confirmed the presence of excessive volumes of free fluid within the pelvis relative to the position of the tip of the child's shunt seen on abdominal X-ray. This ultrasound also confirmed the presence of a hydrocele in the right inguinal region measuring 4x3x3 cm, which was in continuation with free fluid in the pelvic cavity with bowel loops in the vicinity. This was consistent with a patent processus vaginalis, known as a Canal of Nuck and subsequent Canal of Nuck hydrocoele (See Figures 5 & 6). The patient's right inguinal pain and swelling subsided without the need for emergency surgery. However, a plan was made for elective closure of her patent processus vaginalis to prevent future development of similar episodes.

Six months previously this girl was brought to the paediatric emergency department with complaints of abdominal pain and intolerance of gastrostomy feeds. She had abdominal distension and a large mass, approximately 20x20 cm, in the epigastric region. A soft tissue density was evident in the upper abdomen displacing the stomach bubble on abdominal X-ray. Additionally, a departmental ultrasound identified a cyst in the lesser sac containing the tip of the VP shunt, consistent with cerebral spinal fluid (CSF) pseudocyst formation. General surgeons and the neurosurgical team discussed her case. Her management involved draining the cyst and repositioning the distal aspect of the VP shunt back into the peritoneal cavity.

## Discussion

To our knowledge this is the first documented case report of a paediatric Canal of Nuck hydrocoele detected using POCUS. The Canal of Nuck refers to a patent processus vaginalis in females [1]. Embryologically, the processus vaginalis develops around the 12th week of gestation and obliteration of the processus vaginalis normally occurs from the seventh month of gestation to one year of age. The failed closure of the processus vaginalis in females results in continued outpouching of parietal peritoneum through the inguinal canal to the labia majora, known as Canal of Nuck [2]. Passage of intraabdominal contents into this defect is the pathological basis of inguinal hernias in girls. These hernias are most common under 5 years of age but have been reported in girls up to 11-years-old and are associated with prematurity, occurring in 9–11% of premature infants [1,2]. Canal of Nuck hernias often contain bowel and peritoneal fat. For these, ultrasound is the preferred imaging modality [2,3]. They are treated by surgical ligation of the patent processus on an elective basis [4]. However, as with any hernia, contents such as bowel or ovary can become incarcerated and then strangulated and require emergency surgery [3,4]. Where a loculated cyst forms in this region and a hydrocele of the Canal of Nuck exists, its aetiology is generally idiopathic but may be inflammatory or traumatic [5]. Very rarely, a Canal of Nuck hydrocoele may occur as a result of VP shunt dysfunction [6,7]. Owing to its rare nature, a high index of suspicion must be maintained in the appropriate clinical settings such as the presence of VP shunt or known previous complications such as shunt tip migration. Awareness of the clinical presentation of this entity can prevent unnecessary investigations such as computed tomography (CT) being conducted and enable timely and adequate surgical treatment [8,9]. Given the non-specific examination findings in our patient, POCUS was helpful in supporting clinical suspicion that complications from her VP shunt had recurred and caused her symptoms. Specifically, POCUS confirmed that free fluid had migrated from the site of the distal VP shunt catheter into the pelvis and inguinal canal. This was followed by a confirmatory radiology performed ultrasound scan, which was sufficient for a proper preoperative diagnosis of the hydrocele of the Canal of Nuck without the need for ionising radiation.

Peritoneal CSF pseudocyst formation is a rare complication of VP shunts. Previous literature has advocated for the use of CT scans to diagnose CSF pseudocysts [10]. However, owing to the increased susceptibility to harm of irradiating imaging, paediatricians are keen to avoid this wherever possible [11]. In cases where the tip of the VP shunt is required to be visualised within the abdomen, plain X-rays are sufficient to achieve this with a much lower radiation dose compared to an abdominal CT scan [12]. The management of CSF pseudocysts varies based on the presence of shunt malfunction or symptoms. CSF pseudocysts are usually asymptomatic and are incidentally found on imaging. If pseudocysts are not disrupting shunt function or causing abdominal symptoms, they can be monitored conservatively. They can produce symptoms due to mass effect (as happened with this case when compression of the stomach caused intolerance to gastrostomy feeds), bowel obstruction due to adhesions or present purely with neurological features due to compromised CSF flow. CSF cysts are treated by marsupialisation of the cyst (creation of a surgical window) to allow CSF flow back into the general peritoneal cavity along with repositioning the tip of the VP shunt. Recurrence is an ongoing risk.

## Conclusion

We describe the first case of a Canal of Nuck hydrocoele detected using POCUS in a paediatric patient with a VP shunt. As a complication of VP shunt insertion, this is very rare and may be elusive to clinicians due to both a lack of awareness of its existence and because of the symptoms with which they present. Non-specific symptoms such as lower abdominal discomfort pain and nausea may mimic other common paediatric conditions, causing potential delays in diagnosis. This case demonstrated how POCUS can be used to help detect a Canal of Nuck hydrocoele at the bedside promptly, thus reducing delays and minimising radiation exposure from CT scanning for vulnerable patient groups. We recommend POCUS as a safe, reliable first-line imaging tool for Canal of Nuck hydrocoeles in patients with VP shunts and abdominal discomfort.
